# Lithocholic Acid Is an Eph-ephrin Ligand Interfering with Eph-kinase Activation

**DOI:** 10.1371/journal.pone.0018128

**Published:** 2011-03-30

**Authors:** Carmine Giorgio, Iftiin Hassan Mohamed, Lisa Flammini, Elisabetta Barocelli, Matteo Incerti, Alessio Lodola, Massimiliano Tognolini

**Affiliations:** 1 Dipartimento di Scienze Farmacologiche, Biologiche e Chimiche Applicate, Università di Parma, Parma, Italy; 2 Dipartimento Farmaceutico, Università di Parma, Parma, Italy; Semmelweis University, Hungary

## Abstract

Eph-ephrin system plays a central role in a large variety of human cancers. In fact, alterated expression and/or de-regulated function of Eph-ephrin system promotes tumorigenesis and development of a more aggressive and metastatic tumour phenotype. In particular EphA2 upregulation is correlated with tumour stage and progression and the expression of EphA2 in non-trasformed cells induces malignant transformation and confers tumorigenic potential. Based on these evidences our aim was to identify small molecules able to modulate EphA2-ephrinA1 activity through an ELISA-based binding screening. We identified lithocholic acid (LCA) as a competitive and reversible ligand inhibiting EphA2-ephrinA1 interaction (Ki = 49 µM). Since each ephrin binds many Eph receptors, also LCA does not discriminate between different Eph-ephrin binding suggesting an interaction with a highly conserved region of Eph receptor family. Structurally related bile acids neither inhibited Eph-ephrin binding nor affected Eph phosphorylation. Conversely, LCA inhibited EphA2 phosphorylation induced by ephrinA1-Fc in PC3 and HT29 human prostate and colon adenocarcinoma cell lines (IC_50_ = 48 and 66 µM, respectively) without affecting cell viability or other receptor tyrosine-kinase (EGFR, VEGFR, IGFR1β, IRKβ) activity. LCA did not inhibit the enzymatic kinase activity of EphA2 at 100 µM (LANCE method) confirming to target the Eph-ephrin protein-protein interaction. Finally, LCA inhibited cell rounding and retraction induced by EphA2 activation in PC3 cells. In conclusion, our findings identified a hit compound useful for the development of molecules targeting ephrin system. Moreover, as ephrin signalling is a key player in the intestinal cell renewal, our work could provide an interesting starting point for further investigations about the role of LCA in the intestinal homeostasis.

## Introduction

The Eph receptor tyrosine kinases belong to the largest family of tyrosine kinase receptors. To date 16 members, across many species, have been identified [1] and divided into 2 classes (A and B), based on sequence homology of extracellular domain and on their affinity for ephrin ligands. Ephrins are also divided into 2 groups: ephrins A are glycosylphosphatidyl-inositol (GPI)-linked proteins anchored to cell membrane while ephrins B are transmembrane proteins. Ephrins A usually bind to EphA receptors and ephrins B preferentially bind to EphB receptors. Eph-ephrin binding within the same class is highly promiscuous and inter-class binding examples have also been reported [2], [3]. The membrane-bound protein nature of ephrin ligands gives particular features to this system. First of all, cell-cell contact is needed to activate the system, even if ephrins A released or cleaved from cells retain the ability to activate Eph receptors [4], [5], [6]. Second, bidirectional signals are generated by Eph-ephrin interaction: forward signals into the cells expressing Eph receptors go along with reverse signals into the cells bearing ephrin ligands. Finally, increasing evidence shows that not only Eph receptors but also ephrins can transmit signals independently of their interaction, through crosstalk with other signalling pathways [7].

Eph-ephrin system has been extensively studied in embryogenesis where it plays a critical role in tissue boundaries formation and neuronal circuits development [8], [9]. Moreover, several reports have shown an implication of this system in functions like cell growth and survival, cell attachment and migration, highlighting a possible critical role in tumorigenesis, cancer progression, invasiveness and metastasis. Among all Eph receptors, EphA2 is the most widely studied in oncology field because of its expression and function in several cancer types. In fact the EphA2 overexpression results in the transformation of mammary epithelial cells [10] and has been correlated with poor clinical prognosis in many studies [11], [12], [13].

High levels of this receptor have been found in several cancer types including brain, lung, breast, ovarian, prostate, colorectal, and kidney malignancies. Moreover ephrinA1, the physiological EphA2 receptor ligand, is often downregulated when EphA2 is up-regulated and vice versa [6], [14].

For all these reasons EphA2 receptor represents a promising target in cancer therapy and different strategies are under evaluation by several research groups in order to develop specific kinase inhibitors or ligands able to interfere with protein-protein interaction.

Although a first class of small molecule able to antagonize ephrin binding to the EphA4 and EphA2 receptors has been recently identified [15], the ephrin field remains essentially orphan of pharmacological tools able to elucidate its physiopathological role.

With this in mind, we performed an ELISA binding assay screening on an “in-house” chemical library (Table S1). This approach aimed to identify scaffolds that might be utilized to design chemical entities able to inhibit the interaction between EphA2 extracellular domain and ephrinA1. The chemical library includes drugs and endogenous bioactive molecules. The use of drugs is very advantageous because of their already optimized pharmacokinetic and toxicity profiles. On the other hand, the discovery of new activities of physiological, bioactive compounds can provide the basis for novel investigations in pathophysiological fields. In the present work, we describe the discovery of lithocholic acid (LCA), a secondary bile acid, as a novel competitive, reversible antagonist of the Eph-ephrin system.

## Methods

### 1. Reagents

All culture media and supplements were purchased from Lonza. Recombinant proteins and antibodies were from R&D systems. Cells were purchased from ECACC. Leupeptin, aprotinin, NP40, MTT, tween20, BSA and salts for solutions were from Applichem; bile acids, EDTA and sodium orthovanadate were from Sigma. Human IgG Fc fragment was from Millipore (AG714).

### 2. Cell cultures

PC3 human prostate adenocarcinoma cells were grown in Ham F12 supplemented with 7% fetal bovine serum (FBS) and 1% antibiotic solution. HT-29 human colon adenocarcinoma cells were maintained in EMEM supplemented with 10% FBS, 1%NEAA, 1% sodium piruvate and 1% antibiotic solution. T47D human breast tumor cells were grown in RPMI 1640 with 10% FBS and 1% antibiotic solution. All cell lines were grown in a humidified atmosphere of 95% air, 5% CO_2_ at 37°C.

### 3. ELISA screening and Ki/IC_50_ determination

Our chemical collection (Table S1) was stocked in a 20 mM dimethyl sulfoxide (DMSO) solution and we performed binding assay at a concentration of 200 µM. Only compounds displacing more than 40% ephrinA1 from EphA2 receptor were considered for a full concentration-binding curve. 96 well ELISA high binding plates (Costar #2592) were incubated overnight at 4°C with 100 µl/well of 1 µg/ml EphA2-Fc (R&D 639-A2) diluted in sterile PBS (0.2 g/l KCl, 8.0 g/l NaCl, 0.2KH_2_PO_4_, 1.15 g/l Na_2_HPO_4_, pH 7.4). The day after wells were washed three times with washing buffer (PBS +0.05% tween20, pH 7.5) and blocked with 300 µl of blocking solution (PBS +0.5% BSA) for 1 hour at 37°C. Compounds were added to the wells at proper concentration in 1% dimethyl sulfoxide (DMSO) and incubated at 37°C for 1 hour. Biotinylated ephrinA1-Fc (R&D BT602) was added at 37°C for 4 hours at 30 ng/ml in displacement assays or in a range from 1 to 2000 ng/ml in saturation studies. Wells were washed three times and incubated with 100 µl/well Streptavidin-HRP (Sigma S5512) solution (0.05 µg/ml in PBS supplemented with 0.5% BSA, pH 7.4) for 20 minutes at room temperature, then washed again for three times and incubated at room temperature with 0.1 mg/ml tetra-methylbenzidine (Sigma T2885) reconstituted in stable peroxide buffer (11.3 g/l citric acid, 9.7 g/l sodium phosphate, pH 5.0) and 0.02% H_2_O_2_ (30% m/m in water), added immediately before use. The reaction was stopped with 3N HCl 100 µl/well and the absorbance was measured using an ELISA plate reader (Sunrise, TECAN, Switzerland) at 450 nm.

The IC_50_ value was determined using one-site competition non-linear regression and Kd values of the curves with or without antagonists were calculated using one-binding site non-linear regression analysis with Prism software (GraphPad Software Inc.). The K_i_ was obtained using Schild plot [16] where Log[DR-1] is a function of the negative Log10 of the inhibitor concentration. The Hill's coefficient was calculated using linear fitting to evaluate whether the inhibition was competitive or uncompetitive.

### 4. PC3 cell binding

96 well ELISA high binding plates were incubated overnight at 4°C with ephrinA1-Fc 1 µg/cm^2^, 100 µl per well. The plates were washed three times with PBS and blocked for 1 hour with BSA 1%. Cells were treated with proper concentration of substance or DMSO 0.5% for 30 minutes in vials on a shaker. After that, plates were washed with PBS and incubated with 100 µl 5×10^5^ cells/ml for 1 hour at 37°C. Finally, plates were washed with PBS and adhering cells were quantified using MTT colorimetric assay.

### 5. Cell lysates

Cells were seeded in 12-well plates at concentration of 10^5^cells/ml in complete medium until they reached ∼40% confluence and serum starved overnight. The day after cells were treated with compounds under study, vehicle or standard drug, stimulated with the proper agonist, rinsed with sterile PBS and solubilized in lysis buffer. The lysates were resuspended and rocked at 4°C for 30 minutes and then centrifuged at 14000 xg for 5 minutes. The protein content of supernatant was measured with BCA protein assay kit (Thermo scientific), standardized to 200 µg/ml and transferred into a clean test tube ready to be used.

### 6. Phosphorylated-EphA2, -EphB4 and –EGFR

EphA2-, EphB4- and EGFR-phosphorylation were measured in cell lysates using DuoSet®IC Sandwich ELISA (RnD Systems, #DYC4056, #DYC4057 and #DYC1095, respectively) following manufacturer's protocol. Briefly, 96 well ELISA high binding plates (costar 2592) were incubated overnight at room temperature with 100 µl/well of the specific capture antibody diluted in sterile PBS to the proper working concentrations. The day after wells were washed and blocked for 1 hour at room temperature. After that, wells were washed and 100 µl/well of lysates were added at room temperature for 2 hours; wells were washed and incubated with Detection Antibody at room temperature for 2 hours. The phosphorylation was revealed utilizing a standard HRP format with a colorimetric reaction read at 450 nm.

### 7. VEGFR, IRKβ and IGFR1β activity

Stimulant or inhibitory effects of LCA towards IRKβ and IGFR1β activity were tested using alpha Technology (PerkinElmer, Waltham, MA, USA) in HepG2 or A431 cells, respectively. Cells were seeded in microplates at 4×10^4^ cells/well and preincubated for 5 min at 22°C in presence of either HBSS or LCA. Cells were stimulated with 5 nM IGF1 or 100 nM insulin for 10 minutes, lysed and a fluorescence acceptor (alphaLISA protein A beads coated with anti-phospho-IRKβ or -IGFR1β) added for 2 hours. A fluorescence donor (streptavidin coupled-beads) coated with an antibody towards IRKβ or IGFR1β was incubated for 2 hours and the signal was measured at λex = 680 nm and λem = 500 nm and 600 nm using a microplate reader (EnVision, Perkin Elmer, Waltham, MA, USA).

Stimulant or inhibitory activities of LCA towards VEGFR activity were tested in HUVE cells using cellular dielectric spectroscopy. Cells were seeded at 5×10^4^ cells/well into 96-well plate, the following day growth media was exchanged with HBSS buffer +20 mM HEPES and cells were allowed to equilibrate for 75 min with or without 100 µM LCA and stimulated with 0.1 nM VEGF. Impedance measurement was monitored for 10 minutes. VEGF receptor tyrosine kinase inhibitor II (Calbiochem-Merck, Darmstadt, Germany) was used as a standard reference.

All results were expressed as a percent inhibition of the control response to 5 nM IGF1, 100 nM insulin or 0.1 nM VEGF. These assays were performed at CEREP (Celle L'Evescault, France).

### 7. Kinase assay

Evaluation of LCA effects on the kinase activity of human EphA2 was performed by measuring the phosphorylation of the substrate Ulight-TK peptide (50 nM) using the LANCE detection method [17]. Staurosporine was used as reference compound.

### 8. MTT assay

Cell viability was evaluated using the MTT colorimetric assay. Cells were plated in 96-well plates at a density of 10^5^cells/ml and the day after treated with compounds or 0.5% DMSO for 2, 24, 48, or 72 hours. MTT was added at the final concentration of 1 mg/ml and incubated for 2 hours. The resulting formazan crystals were washed with PBS 100 µl/well and then solubilized with DMSO 200 µl/well. The absorbance was measured at 550 nm using an ELISA plate reader and the results were expressed as the ratio between absorbance of the cell treated with the compounds and untreated cells.

### 9. PC3 cell rounding assay

PC3 cells were grown on 12-well culture plates and starved overnight in Ham-F12 medium with 0.5% FBS. DMSO (final concentration 0.25%) or compounds were incubated for 20 min, before stimulation with 0.5 µg/ml ephrinA1-Fc or Fc for 30 minutes. During this time cells were observed and pictures were taken from the same field, before and after incubation, under a microscope (Leica, DM IL). Cell rounding was evaluated using ImageJ program.

## Results

### Lithocholic acid was a competitive and reversible Eph-receptor ligand

To identify compounds interfering with EphA2-ephrinA1 binding we immobilized EphA2-Fc- ectodomain on proper ELISA plates and binding of biotinylated-ephrin-A1-Fc was detected using the colorimetric reaction developed by streptavidine-HRP and tetramethylbenzidine.

Selectivity and specificity of the assay were tested using not-biotinylated ephrin-A1-Fc as a ligand of the EphA2-Fc receptor (Figure S1). As expected, not-biotinylated ephrin-A1-Fc competitively inhibited EphA2-biotinylated-ephrin-A1 binding with a Ki of 102 ng/ml and a Hill coefficient of 1.19. Furthermore, Fc alone did not interfere with the binding process at any concentration.

All the compounds of the chemical collection were incubated for 1 hour at the concentration of 200 µM and only lithocholic acid resulted to significantly reduce EphA2-ephrinA1 binding.

**Figure 1:**
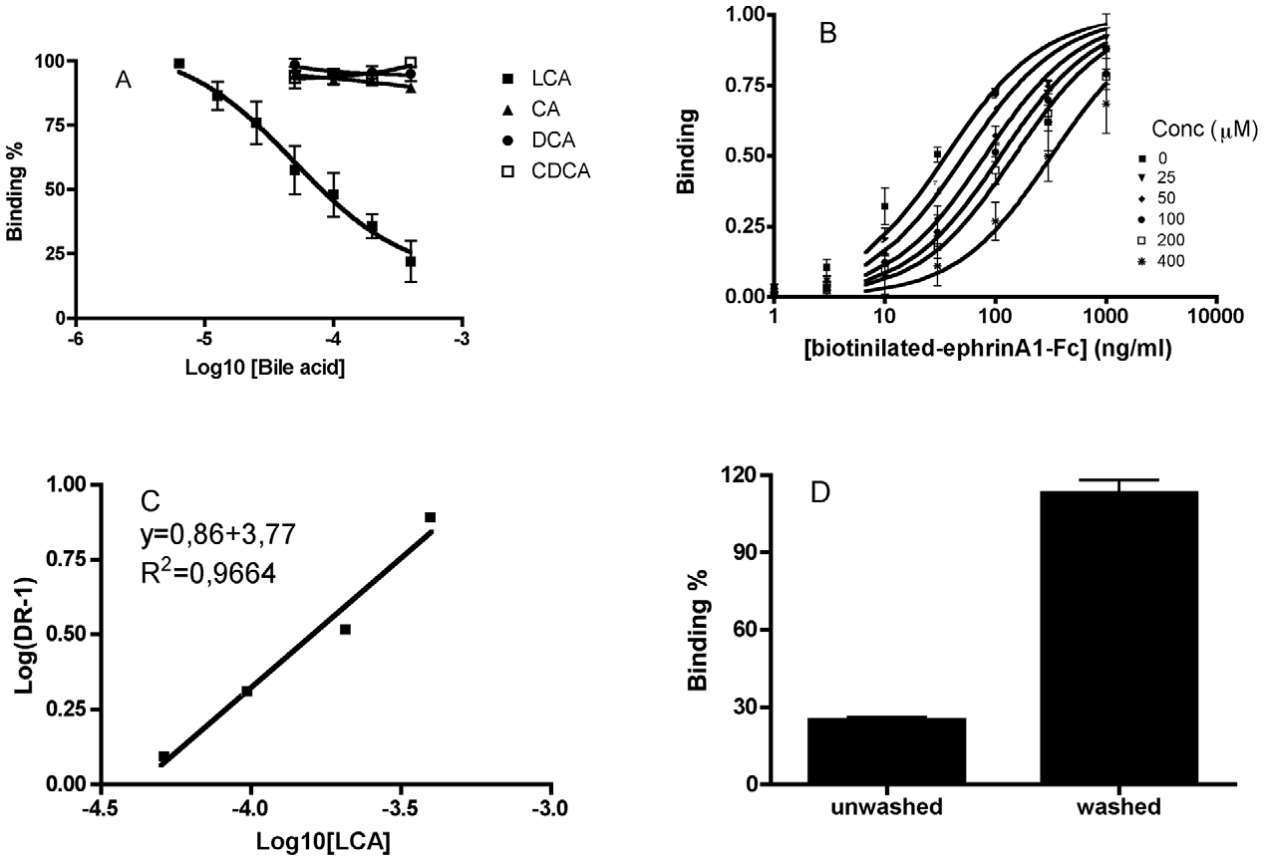
Lithocholic acid competitively inhibited EphA2-ephrinA1 binding. 96 well ELISA high binding plates were incubated O/N with EphA2-Fc and the following day washed and blocked with PBS +0.5% BSA for 1 hour at 37°C. Compounds were added in the wells at proper concentrations 1 hour before the addition of biotinylated ephrinA1-Fc. After 4 hours wells were washed and incubated with a streptavidin-HRP solution for 20 minutes at room temperature. Wells were washed again and incubated with tetra-methylbenzidine. The reaction was stopped with 3N HCl and the absorbance was measured at 450 nm. A, lithocholic acid dose-dependently displaced binding of ephrin-A1-Fc ectodomain from immobilized EphA2-Fc ectodomain. B, binding of ephrin-A1-Fc ectodomain to immobilized EphA2-Fc ectodomain in presence of different concentration of lithocholic acid. C, The dissociation constants (Kd) from the previous plot were used to calculate Log (Dose-ratio - 1) and to graph the Schild plot. pKi of lithocholic acid was estimated by the intersection of the interpolated line with the X-axis. The slope of the interpolated line can be related to the nature of the binding. A slope between 0.8 and 1.2 is related to a competitive binding whereas higher numbers are related to non-specific interactions. D, EphA2-ephrinA1 binding in presence of 200 µM LCA with or without washing three times with PBS.

We repeated the experiment testing LCA together with bile acid analogues: cholic (CA), deoxycholic (DCA) and chenodeoxycholic (CDCA). CA, DCA and CDCA did not displaced EphA2-ephrinA1 binding whereas LCA decreased it by 68.2%±4.3. In order to calculate the inhibitory concentration reducing binding of 50% (IC_50_) we charted a displacement curve using increasing concentration of LCA (in the range of 12.5–400 µM) towards biotinylated-ephrinA1-Fc at a concentration corresponding to its K_D_. In these conditions we obtained a dose-dependent displacement and we calculated a pIC_50_ of 4.24±0.068 (corresponding to an IC_50_ of 57 µM, Figure 1A). To evaluate the nature of the antagonism we plotted saturation curves of EphA2-ephrinA1 binding in presence of increasing concentrations of LCA (Figure 1B). We calculated the K_D_ or the apparent K_D_ of each curve and we drew a Schild plot, where Log[DR-1] is a function of the –Log10[inhibitor] [16] (Figure 1C). We obtained a well-interpolated regression line (r^2^ = 0.9664) having a slope of 0.8618. A slope between 0.8 and 1.2 is associated with a competitive binding. Finally, the pKi resulting from the intersection of the interpolated line with the X-axis resulted to be equal to 4.31±0.03 (corresponding to a Ki of 49 µM). We repeated displacement experiments incubating 200 µM LCA for 1 hour and washing some wells before adding 50 ng/ml ephrinA1-Fc. The displacement was detected only where the washing was not performed, suggesting the reversibility of the LCA binding to EphA2-ephrinA1 system (Figure 1D).

**Figure 2:**
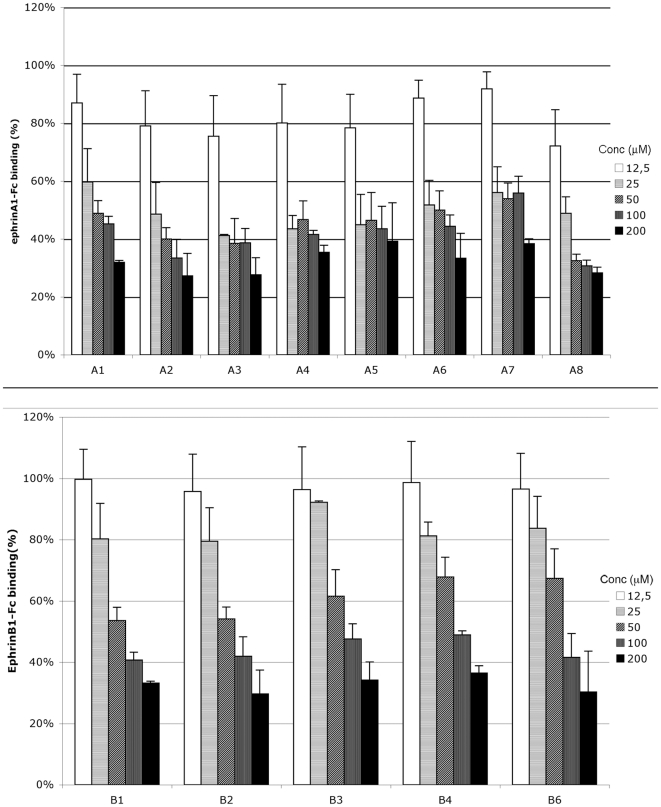
Lithocholic acid did not discriminate Eph-kinases subclasses. A, lithocholic acid dose-dependently displaced binding of ephrin-A1-Fc ectodomain from immobilized EphA-Fc ectodomains. B, lithocholic acid dose-dependently displaced binding of ephrin-B1-Fc ectodomain from immobilized EphB-Fc ectodomains. Data are the means of at least three independent experiments ± st. err.

Next, we tested LCA activity towards all the EphA and EphB kinases using biotinylated ephrinA1-Fc and biotinylated ephrinB1-Fc, respectively, at their K_D_ concentration. LCA showed to be a promiscuous ligand of EphA and EphB receptor subfamilies, suggesting the existence of a common mechanism of interference towards Eph-ephrin binding (Fig 2).

**Figure 3:**
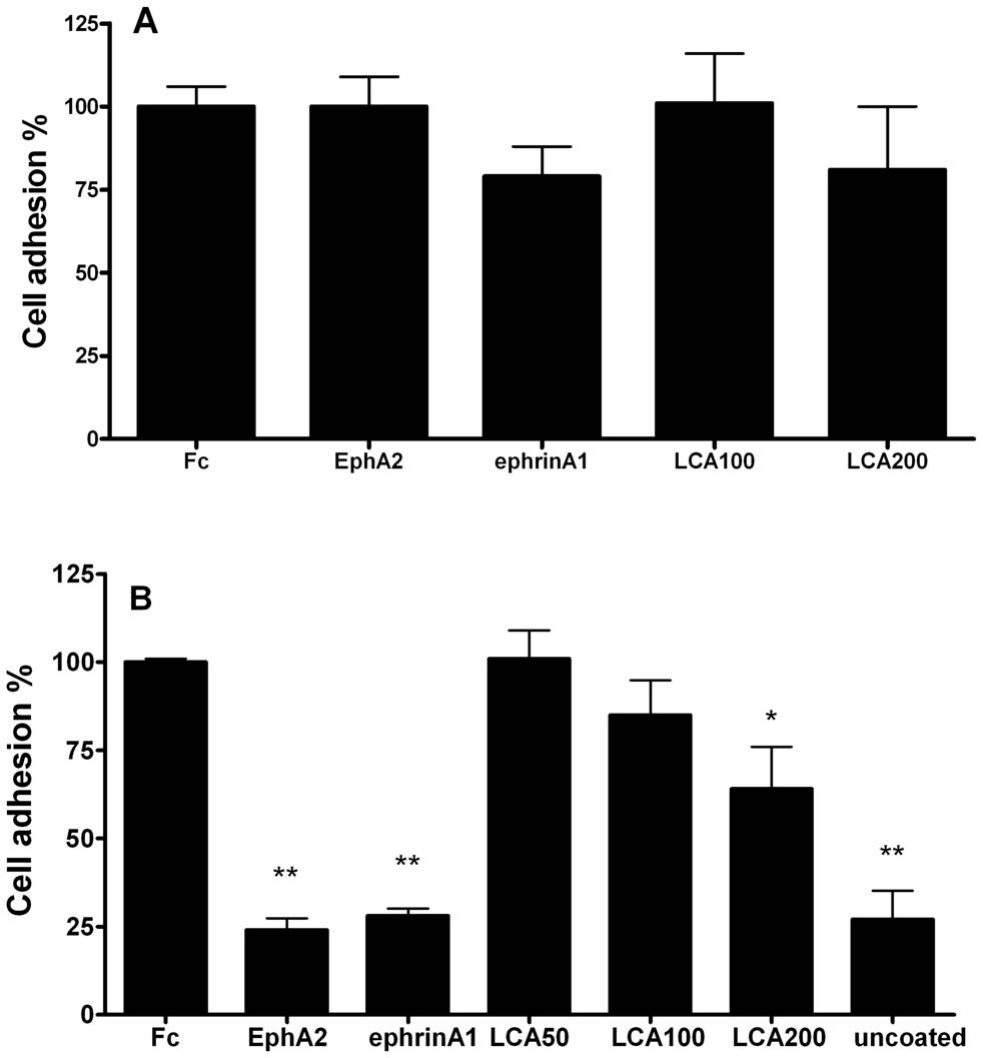
Lithocholic acid dose-dependently inihibited PC3 adhesion to ephrinA1-Fc. 96-wells plates for cell culture were untretaed (A) or coated with 1 µg/cm^2^ ephrinA1-Fc (B) overnight. PC3 cells were treated with the indicated compounds for 30 minutes in a tube and let to adhere for 60 minutes on the wells. Cell adhesion is reported normalizing adhesion of Fc to 100%. LCA concentrations are reported as µM. Data are the means of at least three independent experiment ± st. err. One-way ANOVA followed by Dunnet's post test was performed comparing Fc to all other column. No significant differences were detected for data in graph A. *, p<0.05, **,p<0.01.

We also simulated *in vivo* conditions immobilizing ephrinA1-Fc on high binding plates and performing adhesion with PC3 cells. In these experiments ephrinA1-Fc effectively mediated cell adhesion through EphA2-ephrinA1 interaction. In fact, preincubation with 4 µg/ml EphA2-Fc or ephrinA1-Fc completely abolished PC3 adhesion. Similarly, LCA dose-dependently inhibited PC3 adhesion to ephrinA1-Fc (Fig 3B). The experiment was repeated on uncoated standard cell culture plates where aspecific adhesion was mediated by multiple factors (selectins, integrins, cadherins) and not by Eph-ephrin interaction [18]. Figure 3A reports that neither ephrinA1-Fc nor LCA inhibited aspecific cell adhesion. However, a discrepancy between affinity of LCA in binding assays and its potency in adhesion study is noticed. Such a divergence could be related to the differences between the ELISA-binding study and the functional adhesion study due to, incubation times (1 hour +4 hours for binding; 30 minutes +1 hour for adhesion), biological system (only proteins for binding; cells and proteins for adhesion) and sensitivity of the revelation method (HRP for binding; MTT for adhesion).

### LCA acid inhibited Eph-kinases phosphorylation at not-cytotoxic concentrations

Functional studies were performed in cultured cells to evaluate agonist or antagonist properties of LCA and other bile acids at Eph receptors. We used PC3 human prostate adenocarcinoma and HT29 human colon adenocarcinoma cells as a model for their known ability to naturally express EphA2 [18], [19]. Moreover, PC3 are a well established model to study Eph-ephrin pharmacology whereas HT29 cells are commonly used to study the physiological role of bile acids [20].

In these studies we stimulated EphA2 phosphorylation with 0.25 µg/ml ephrinA1-Fc on PC3 or HT29 cells, in presence or absence of bile acids. Dasatinib 1 µM was used as reference compound being a multikinase inhibitor endowed with a high potency towards Eph kinases [21].

**Figure 4:**
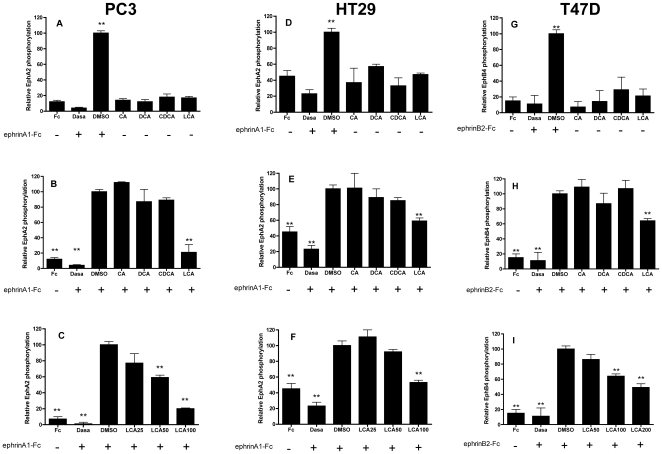
Lithocholic acid dose-dependently inhibited Eph-kinases phosphorylation. EphA2 phosphorylation was induced by 0.25 µg/ml ephrinA1-Fc in PC3 (A, B, C) or HT29 cells (D, E, F). EphB4 phosphorylation was stimulated with 3 µg/ml ephrinB2-Fc, preclustered with 0.3 µg/ml IgG Fc fragment on T47D cells (G, H, I). Cells were pretreated for 20 minutes with 1% DMSO, 100 µM bile acids or the indicated concentrations (µM) of LCA and stimulated for 20 minutes with ephrinA1/B2-Fc (+) or Fc alone(−) as a control. Phospho-EphA2/B4 levels are relative to ephrinA1/B2-Fc+DMSO. Data are the means of at least three independent experiment ± st. err. One-way ANOVA followed by Dunnet's post test was performed comparing Fc to all other columns for Fig A, D, G and ephrinA1-Fc+DMSO to all other columns for Fig B,C,E, F, H, I. *, p<0.05, **, p<0.01.

Consistently with binding studies, 100 µM CA, DCA and CDCA were inactive towards Eph kinases phosphorylation both when studied as agonist (Fig 4A, D) or antagonists (Fig 4B, E). On the other hand data in figures 4A-F demonstrated that LCA is an EphA2 antagonist inhibiting in a dose-dependent manner ephrinA1-Fc induced EphA2 phosphorylation with an IC_50_ = 48 µM and 66 µM on PC3 and HT29 cells, respectively.

As LCA showed to be a promiscuous ligand of EphA and EphB receptor subfamilies we tested LCA activity against EphB4 phosphorylation on T47D breast cancer cells induced by 3 µg/ml ephrinB2-Fc, preclustered with 0.3 µg/ml of IgG Fc fragment. LCA dose-dependently inhibited EphB4 phosphorylation with an IC_50_ of 141 µM(Figure 4G–I). This value is higher than the value obtained for EphA2 phosphorylation and it is consistent with binding data where LCA has a 2-fold lower affinity towards EphB receptors when compared to EphA receptors. MTT assay demonstrated that concentrations tested in phosphorylation studies were not cytotoxic (Figure S2).

### LCA antagonized Eph-kinase phosphorylation inhibiting protein-protein interaction

**Figure 5:**
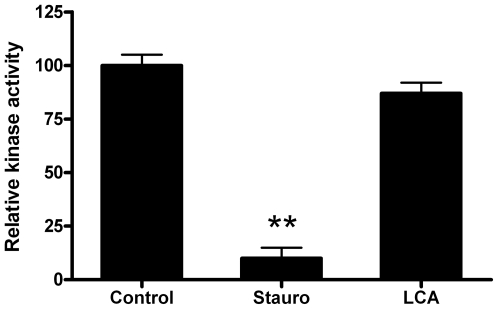
Lithocholic acid did not modify EphA2 enzymatic activity. Recombinant human EphA2 enzyme activity was evaluated with LANCE® method using ATP and Ulight-TK peptide as substrate (http://las.perkinelmer.com/). Human EphA2 kinase was previously incubated with 100 µM LCA, 1 µM staurosporine or 1% DMSO (control) for 30 minutes. T-test was performed comparing LCA and staurosporine to control. **,p<0.01.

In order to exclude a direct inhibition of LCA with Eph kinase domain, an enzyme-based assay was performed. Briefly, incubation of recombinant EphA2-kinase induced the phosphorylation of a proper substrate (Ulight-TK peptide 50 nM) which was recognized by an Europium-labeled anti-phospho antibody and resulted in light emission (LANCE detection method, [17]). Incubation of the protein with staurosporine, used as reference compound, inhibited kinase activity (IC_50_ = 93 nM), whereas incubation with LCA up to 100 µM did not modify enzymatic activity (Fig 5).

### LCA did not affect EGFR, VEGFR, IRKβ or IGFR1β activities

**Figure 6:**
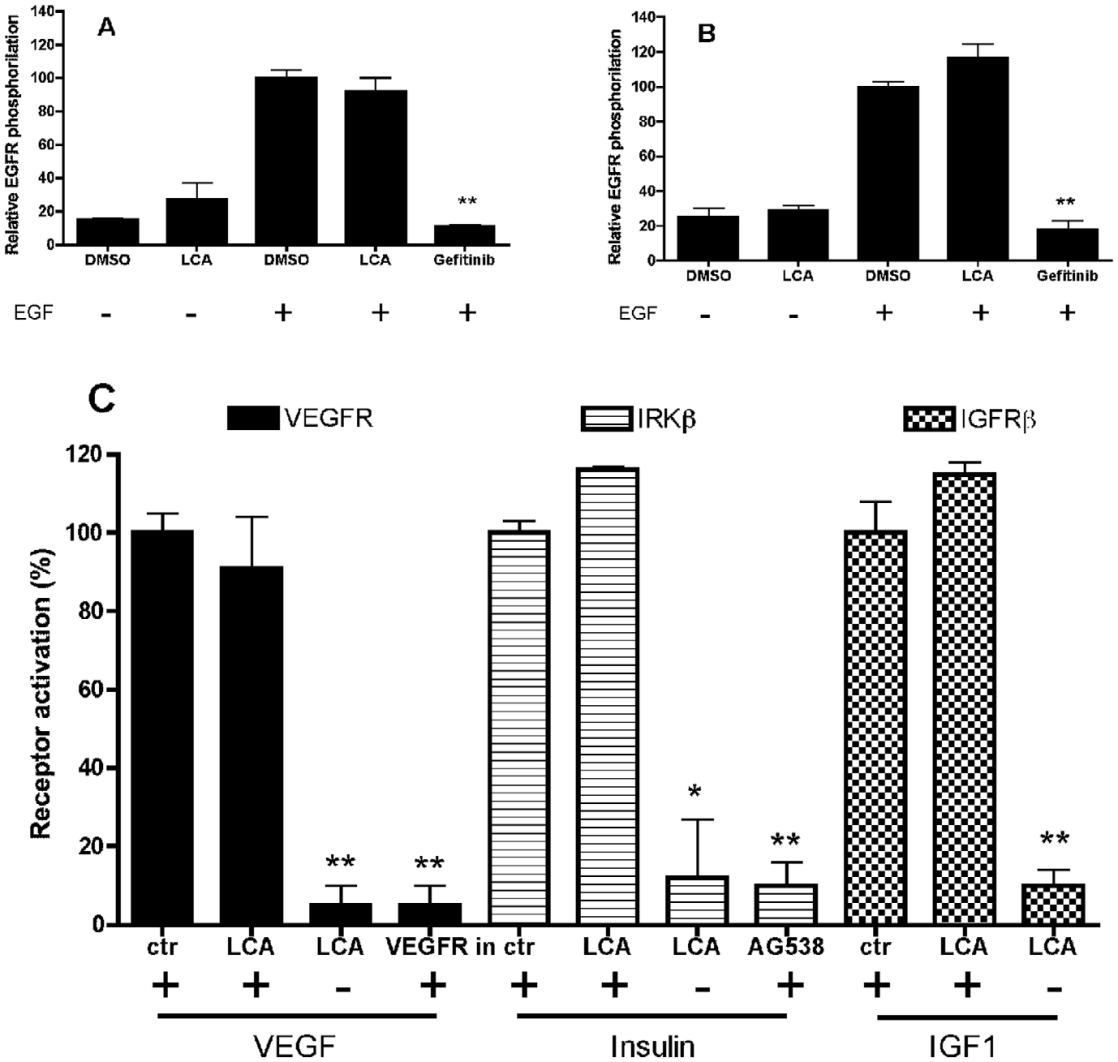
Lithocholic acid did not affect EGFR, VEGFR, IRKβ or IGFR1β activity. A, B) EGFR phosphorylation was induced by 30 ng/ml and 10 ng/ml EGF on PC3 (A) and HT29 (B) cells, respectively. Cells were pretreated for 20 minutes with 1% DMSO, 100 µM LCA or 10 µM gefitinib and stimulated for 20 minutes with EGF. Phospho-EGFR levels are relative to EGF+DMSO. Data are the means of at least three independent experiments ± st. err. T-test was performed comparing Fc to LCA and EGF+DMSO to EGF+LCA and EGF+gefitinib. **,p<0,01. C) HUVE, HepG2 or A431 cells, were stimulated for 10 minutes with 0.1 nM VEGF, 5 nM IGF1 or 100 nM insulin, respectively, in presence of 100 µM LCA or the proper inhibitor as a reference (1 µM VEGFR inhibitor II or 10 µM AG538). Data are the means of two experiments ± st. err. T-test was performed comparing ctr to other column of the same receptor. **,p<0.01.

In order to assess the specific interaction of LCA with Eph-kinases we performed functional assays on other receptor tyrosine kinases (EGFR, VEGFR, IRKβ or IGFR1β). LCA 100 µM was completely inactive when tested towards the phosphorylation of EGF receptors induced by EGF both in PC3 and HT29 cells (Fig 6A,B) whereas the EGFR kinase inhibitor gefitinib (10 µM), used as reference compound, completely abolished response to EGF. Similarly LCA failed to affect activity of VEGFR, IRKβ or IGFR1β both when tested as an agonist or an antagonist (Fig 6C).

### LCA antagonized EphA2-dependent PC3 cell rounding

**Figure 7:**
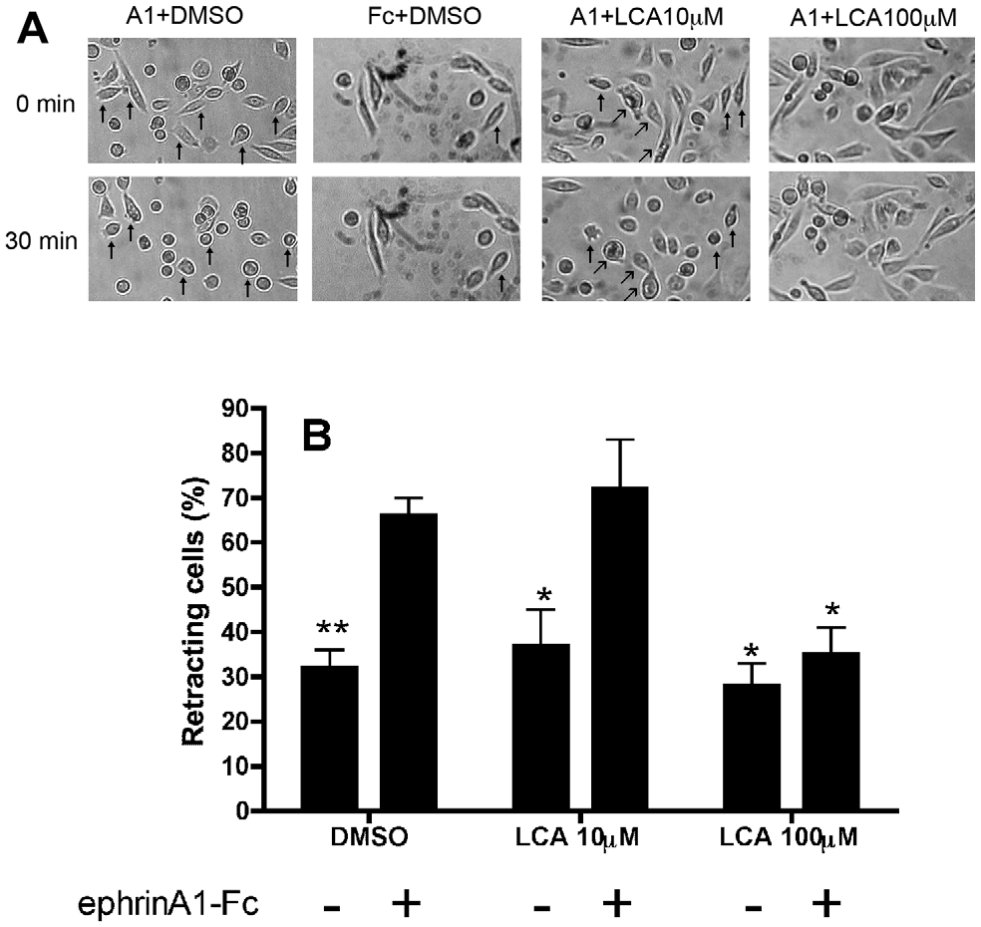
Lithocholic acid antagonized EphA2 dependent PC3 cell rounding. Serum starved PC3 cells were stimulated with 0.5 µg/ml ephrinA1-Fc or Fc for 30 minutes in presence of DMSO or LCA preincubated for 20 minutes. A, Morphological changes of PC-3 cells induced by ephrinA1-Fc or Fc treatment in presence of LCA 100 µM, LCA 10 µM and DMSO 0.25% added 20 minutes before. Cell images were collected from the same field at time 0 and 30 minutes using a digital camera mounted on a Leica DM IL microscope. B, Histogram showing the average percentage of retracting cells 30 minutes after a treatment with ephrinA1-Fc or Fc in presence of LCA 100 µM, LCA 10 µM and DMSO 0.25% preincubated for 20 minutes. Cells, which rounded their shape and having an area less than 20% of the initial value, were scored as retracting. Data are the means of at least three independent experiments ± st. err. One-way ANOVA followed by Dunnet's post test was performed comparing ephrinA1-Fc+DMSO to all other columns. *, p<0.05, **,p<0.01.

Previous studies showed that PC3 cells express mainly EphA2 receptors and their activation lead to cell retraction and rounding [18]. To assay the antagonistic properties of LCA we examined whether LCA was able to inhibit PC3 cell rounding induced by 0.5 µg/ml ephrinA1-Fc. The results (Fig 7) showed that LCA blocked cell rounding at 100 µM, concentration inhibiting completely EphA2 phosphorylation, while it was inactive at 10 µM, subthreshold concentration towards EphA2 phosphorylation. Neither DMSO 0.25% nor LCA alone induced changes in cell morphology when incubated with Fc for 30 minutes.

## Discussion

In the present work we showed for the first time the interaction of lithocholic acid (LCA), a secondary bile acid, with Eph-ephrin system. We demonstrated that LCA caused a reversible and competitive displacement of the biotinylated ligand ephrinA1-Fc from the receptor EphA2-Fc in cell-free binding studies and we pointed out the antagonistic properties of LCA in phosphorylation studies in different cell lines.

Bile acids had been considered for long time only as detergent molecules necessary for lipid solubilization and absorption in the intestine during digestion. However, many studies have explored the hypothesis that bile acids also work as regulatory molecules. A recent paper [20] used bile acid enantiomers to differentiate their receptor- and non-receptor-mediated effects in HT29 and HCT116 colon cancer cells. It definitely proved that bile acid-induced cytotoxicity and apoptosis is enantiospecific and correlates with a receptor interaction rather than aspecific detergent properties. Other papers described specific interaction of bile acids with the nuclear farnesoid X receptor, mainly involved in hepatic lipid and glucose metabolism [22] and with the G protein coupled receptor TGR5 whereby they induce intracellular cAMP increase in CHO cells [23].

Consistently, our work suggests that LCA can act through the interaction with specific receptors. In fact we provided evidence of a competitive antagonism towards Eph-ephrin binding. First of all we obtained the proper displacement of saturation curves, the proper slope of the Schild plot and the reversibility of the binding. In second place LCA inhibited Eph-kinase phosphorylation induced by ephrinA1-Fc on PC3 and HT29 cell lines but it did not affect enzymatic activity confirming to target the Eph-ephrin protein-protein interaction. Conversely, LCA was inactive against other RTKs such as EGFR, VEGFR IRKβ, IGFR1β in cellular functional studies demonstrating that LCA interfered neither with kinase domain nor with protein-protein interaction of these RTKs. Moreover, the Ki of LCA towards EphA2-ephrinA1 interaction was six times lower than its critical micelle concentration [24], LCA was devoid of any toxicity at the studied concentrations and both binding and functional tests reported the same range of LCA activity included between 20 to 100 µM. Finally, structurally related bile acids bearing only minor chemical modifications on position 7-OH (CDCA), 12-OH (DCA) or 7- and 12-OH (CA) were completely inactive both in binding and phosphorylation studies.

Since Eph–ephrin binding is highly promiscuous also LCA does not discriminate Eph-receptor subclasses A and B. Therefore we can speculate an interaction with a highly conserved region essential for both EphA and EphB receptor binding to their physiological ligands. Ephrin recognition by Eph receptors is mediated by ephrin G–H loop (key) that inserts into a hydrophobic Eph receptor channel shaped by D–E and J–K loops (lock). [25]. Taken together these evidences suggest an interference of LCA with the proper full insertion of the ephrin G–H loop into the Eph-receptor hydrophobic channel. Structural studies will be essential to clarify the dynamic of this interaction.

LCA interfered with Eph-ephrin protein-protein interaction with a higher affinity towards ephrin A system than ephrin B system. Functional studies, carried out on different cell lines, showed that LCA is an antagonist of Eph receptors, because it was able to inhibit the phosphorylation of both EphA2 and EphB4 receptors when stimulated with ephrinA1-Fc and ephrinB2-Fc, respectively. Notably, in accordance to binding studies, LCA showed to have a higher efficacy in inhibiting EphA2 phosphorylation than EphB4 phosphorylation.

The inhibition of Eph-ephrin system could be very useful in the regulation of tumor progression. In fact, several studies highlighted an important role for EphA2-ephrinA1 and EphB4-ephrinB2 interaction in tumor angiogenesis [7]. Furthermore, EphA2 or EphB4 inhibition could reduce ameboid-type migration of cancer cells and could stabilize epithelial adherens junctions in various cancer cell lines, as suggested by Fang and Yang [26], [27].

Moreover, the present work showed that LCA was able to inhibit cell rounding and retraction in PC3 cell line upon EphA2 stimulation, suggesting that this molecule can actually antagonize the effects mediated by EphA2. The inhibition of EphA2 could be advantageous in cancer therapy whenever EphA2 activation mediates tumor progression as previously demonstrated on mammary tumors and melanoma cells [28], [29]. Currently, many strategies to block EphA2 signaling have been explored. Inhibition of EphA2 activation by soluble EphA receptors, binding with antibody or downregulation with siRNA resulted in decrease of cell adhesion, angiogenesis, tumor growth and metastasis, demonstrating that EphA2 may be an important target for anti-tumorigenic and anti-angiogenic therapies [30], [31], [32]. High affinity peptides binding to EphA2 receptors were identified by means of a phage library screening. Binding peptides shared the φxxφ motif where φ is an aromatic amino acid and x is a non-conserved amino acid [33]. Unfortunately, peptides, siRNA and antibodies are quite hard to use in any human therapy because of their very unfavorable pharmacokinetic and pharmacodynamic profiles. On the other hand, low molecular weight ligands still represent a very valuable way to produce and administer drugs. For this reasons our discovery can be a starting point for future research aimed at the development of Eph-ephrin targeting molecules. In fact modulation of the pharmacophore elements present in bile acids could provide high affinity binding molecules, as previously testified by the development of TGR5 and FXR agonists [34].

In addition to the pharmacological features our discovery suggests intriguing pathophysiological implications. In fact, the expression levels of Eph-receptors and ephrin-ligands have a critical role in the organizing cell renewal of the intestine [35], [36]. As lithocholic acid fecal concentration is about 2 mM [37], it is reasonable to suppose an involvement of this secondary bile acid with ephrin system signaling *in vivo*. Consequently, LCA could play a role in the intestinal homeostasis and an alteration of its physiological amount could modify the expression and the signaling of ephrin receptors and ligands. In this way the correct segregation, proliferation and differentiation mechanisms, underlying the tissue homeostasis, could be alterated. Therefore, our findings could be useful for further studies aimed to explain the correlation between the concentration of fecal secondary bile acids (mainly DCA and LCA) and the colorectal cancer incidence, highlighted by several epidemiology studies [38], [39], [40], but whose molecular mechanisms are far to be clear.

## Supporting Information

Table S1 List of the compounds used in the ELISA-based binding study.
(PDF)

Figure S1 EphrinA1-Fc competitively displaced biotinylated-ephrinA1-Fc binding to EphA2. The Calculated Ki was 102 ng/ml and the Hill slope was 1.19.
(PDF)

Figure S2 Cytotoxicity of bile acids on PC3, HT29 and T47D cells after 2 hours of incubation with the indicated compounds. Data are means±SEM.
(PDF)
