# Supplementary material for: Lithocholic Acid Is an Eph-ephrin Ligand Interfering with Eph-kinase Activation
Source: PLoS One. 2011 Mar 30;6(3):e18128. doi: 10.1371/journal.pone.0018128 (PMC3068151; doi:10.1371/journal.pone.0018128)
Supplement: Table S1 — List of the compounds used in the ELISA-based binding study. (PDF) [file pone.0018128.s001.pdf]

List of the compounds used in theELISA-based binding study.

Acetylsalicylic acid	Clavulanic acid	L-citrulline	Pilocarpine
Acetylthiocholine	Clonidine	Levulinic acid	(+) - Pinoresinol
ADP	Clozapine	L-glutamic acid	Piracetam
Alcian blue	p-Coumarinic acid	L-histidine	Pirenzepine
Alpha fluor methyl histidine	Creatinine phosphate	Lidocaine	Potassium methane sulfonate
alpha-Bisabolol	Cromolyn	Linalool	Prazosin
Aminguanidine bicarbonate	Daidzein	Lithocholic acid	Procaine
Aminguanidine hydroxy chloride	Damascone	L-Tartaric acid	Proglumide
4-aminobutyric acid	Deoxycholic acid	L-Tyrosine	Promethazine
5-Aminovaleric acid	2-Deoxyglucose	Luminol	Propranolol
4-Aminopyridine	Dexamethasone	Luteolin	Purpurogallin
Antipyrine	DF 545	Malic acid	Pyrilamine
2-APB	DF 594	D-Mannitol	Pyrogallic acid
Arginine	Diazepam	D(+)-Melezitose	Quercetin
Ascorbic acid	Dimaprit	Methoctramine	Ranitidine
Atenolol	2,3-Dimethylindole	Methyl histamine	Ranitidine hydroxy chloride
ATP	Donepezil hydroxy chloride	2-methyl-5-HT	Reserpine
Atropine	2,2-diphenyl-1-pycrilhydrazyl	Methyl orange	RP 73401
Avertin	Dopamine	methylene blue	Rutin
7-Azaindole	DPCPX	3-methylindole	Salbutamol
Azathioprine	DPPH	4-Methylumbelliferone	Salicylic acid
Azelaic-acid	EDTA	Methysergide	Serotonin
Benzenesulfonamide	EGTA	Metiamide	Sinapinic acid
Benzisothiazol	(-)-Epicatechin	Myristic acid	Impromidine
Beta-escin	(+)-Epicatechin	NAD	Sodium borate
Beta-Gentiobiose	Epinephrine	Naloxone hydroxy chloride	Sodium nitroprussiate
Bethanecol	Erythro-9-(2-hydroxy-3-nonyl)adenine	Naphazoline	Sodium taurocholate
Betulinol	Eugenol	Naringin	Spinaceamin
Borneol	Famotidine	Nerol	Streptomycin
Bromophenol blue	Ferulic acid	Nesosteine	Suberic acid
Burimamide	Gallic acid	Nifedipine	Sulglycotide
Buspirone	Genistein	n-isopropyl epinephrine	Sulphanilamide
2,3-Butanedione monoxime	Geraniol	Nitrobenzylthioinosin	Sulphanilic acid
Caffeic acid	Gibberellic acid	Nitrotetrazolium blue chloride	Sulpiride
Caffeine	Guanethidine	Nw-nitro-L-arginine-methyl ester	Suramin
Carbamoylcholine chloride	Hesperidin	1-Octacosanol	Sylimarin
Carbenoxolone	Hexamethonium	Octatropine methylbromide	Tacrine
Carvacrol	Histamine	Orthovanadate	Theobromine
(-)-trans-Caryophyllene	3-Hydroxytyramine hydrochloride	Oxlate Calcium	Theophylline
Chenodeoxycolic acid	HTAB	Oxmetidine	Thidiazuron
1-(2-chlorophenyl)-3-(dimethylamino)-1-phenyl-prapan-1-ol	Hypoxanthine	Oxybutynin hydrochloride	Thiobarbituric acid
Chlorpheniramine	Ibuprofen	Papaverine hydrochloride	Thioperamide
Chlor-trimeton maleate	Imidazole	Paracetamol	Thymol
Cholestan	Imipramine	para-Nitrophenylphophate	Tiotidine
5-alpha-Cholestan-3-beta-ol	Indigo carmine	Pelargonaldehyde	Trehalose

Cholesterol	Indole	Phenazine methosulfate	Tripelennamine
Cholic acid	Indomethacin	Phenolphthalein	Tyramine
Choline chloride	Inositol	S(+)-N6-(2-phenyl-isopropyl)adenosine	Ursodeoxycholic acid
Cimetidine	Ipratropine	Phenylbutazone	Valeraldehyde
Cinnamaldehyde	Isoprotenerol	Phenylephrine hydrochloride	Verapamil
Cinnamic acid	Kanamycin sulfate	2-Phenylindole	Xylitol
Citric acid	Ketanserin	Physostigmine	Yohimbine
