# Supplementary figures and images for: Lithocholic Acid Is an Eph-ephrin Ligand Interfering with Eph-kinase Activation
Source: PLoS One. 2011 Mar 30;6(3):e18128. doi: 10.1371/journal.pone.0018128 (PMC3068151; doi:10.1371/journal.pone.0018128)

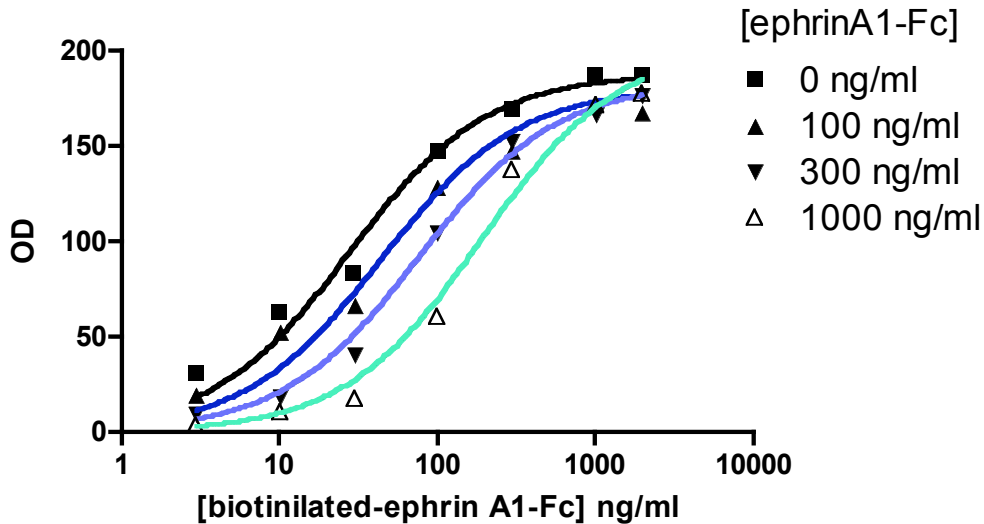

Supplement: Figure S1 — EphrinA1-Fc competitively displaced biotinylated-ephrinA1-Fc binding to EphA2. The Calculated Ki was 102 ng/ml and the Hill slope was 1.19. (PDF) [file pone.0018128.s002.pdf]

**Cytox 2h PC3**

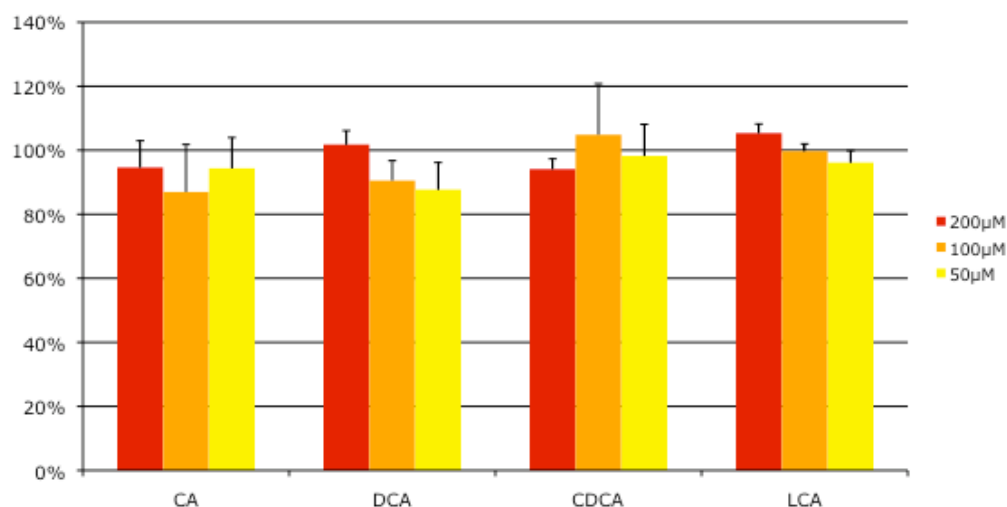

**Cytox 2h HT29**

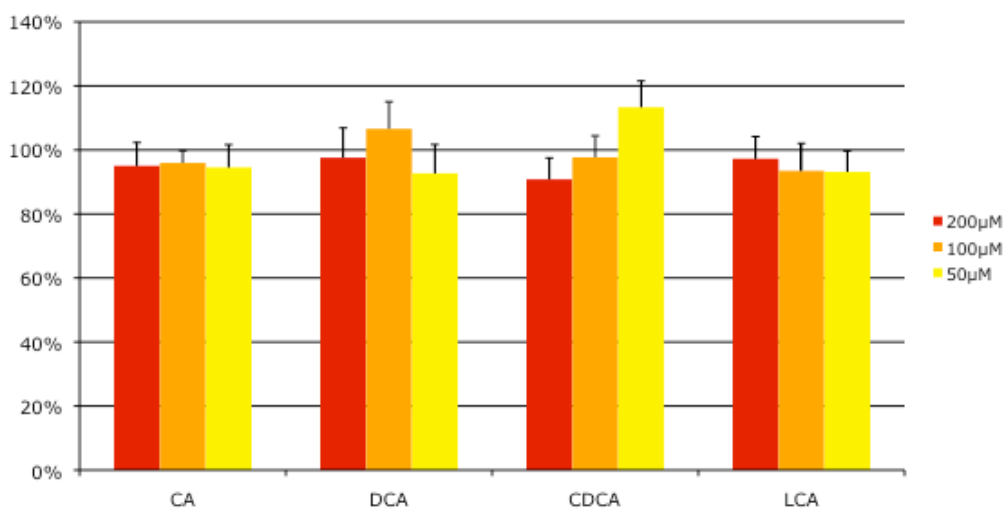

**Cytox 2h T47D**

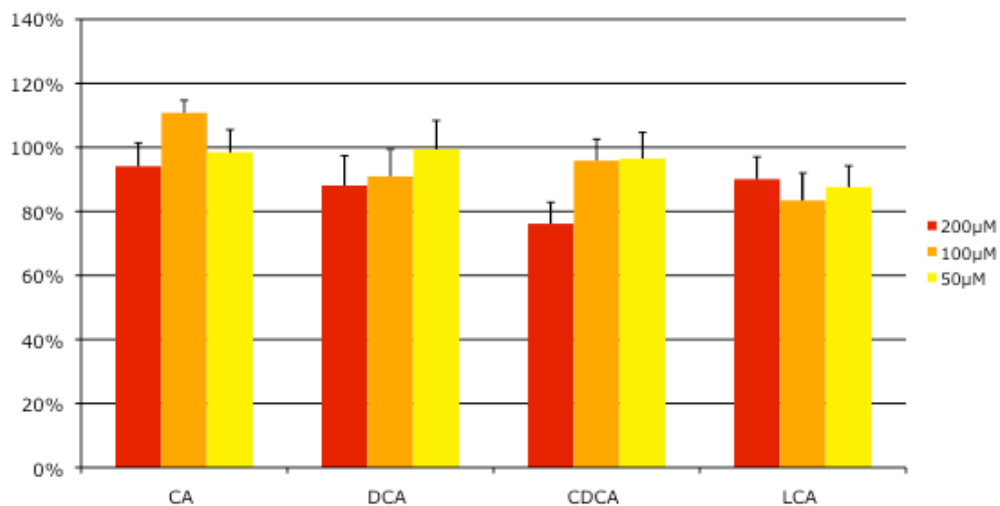

Supplement: Figure S2 — Cytotoxicity of bile acids on PC3, HT29 and T47D cells after 2 hours of incubation with the indicated compounds. Data are means±SEM. (PDF) [file pone.0018128.s003.pdf]
